# Mr. Hope's Case of Extra-Uterine Conception

**Published:** 1801-10

**Authors:** T. Hope


### *To the Editors of the Medical and Phyſical Journal.*

GENTLEMEN,

I Have tranſmitted the following ſingular, and to me extraordinary caſe, that if it be thought of importance ſufficient to occupy a corner of your valuable Miſcellany the inſertion will oblige, Your's, &c.

T. HOPE.

---

On the 2d of Auguſt, 1801, I was called in to attend the wife of —— Shotten, in the pariſh of Rainham, a ſpare ſmall woman of about thirty years of age, who had about three weeks weeks before been delivered of a child, by an accoucheur employed for that purpofe.

The appearance of my patient at the firft fight gave me very unfavorable impreffions, which were more ftrongly confirmed by an enquiry into the hiftory of her fituation from the firft. The following is the fubftance of the account I received.

That in the month of April, 1800, being feven months advanced in pregnancy, fhe was attacked with fevere labor pains. Expecting fhe was going to mifcarry, fhe fent for her family furgeon, who attended her fome time; thefe pains however, although pretty ftrong, and continuing for three weeks, were not fufficient to expel the fœtus, at the end of which period the pains fubfided, but from the low ftate to which fhe was reduced, kept her bed for four weeks after, during all which time fhe had fœtid, irregular menftruation. The fymptoms of labor being entirely removed, fhe complained of an exceeding hard, painful body, adhering to her right fide, and which fubftance fhe never after could get removed, but continued hard, large, and painful.

In the month of November following, fhe difcovered fymptoms of recent impregnation, and was delivered of a child in July laft, of rather an unhealthy appearance; after delivery, the pain and hardnefs of her fide gave her particular uneafinefs, of which fhe complained to the laft moment of her life; other circumftances went on as regular and well as is cuftomary in parturient cafes, until the end of near a fortnight, when having been coftive for fome days, a medicine was given her to procure a ftool, which fhe threw off her ftomach, and foon after every thing elfe that was given.

On the 30th of July, her vomitings were mixed with ftercoraceous matter, as well as the matter of glyfters injected per anum; which induced them to call me in, but which could not be remedied, as nothing, of however light a form or fedative power, could be retained an hour on the ftomach; yet fhe continued in exiftence until the 9th of Auguft.

Having obtained permiffion, two medical friends went with me to open the body. On cutting through the peritoneal lining, we found the hard fubftance of which fhe had fo long complained, to be the uterus, of about the fize of a calf's bladder when diftended with air, and perfectly green from putrefaction. Wifhing to remove it entire, if poffible, as the fubftance was exceedingly thin, we were particularly cautious; yet with all attention, the cavity was broken into, from whence iffued about a pint or more of nafty thick matter, of the confiftence of molaffes, and colour of ftrong beer grounds, in which was a fœtus of between fix and feven months, fo macerated that it had

 fcarcely

ſcarcely ſufficient ſolidity of bone to afford reſiſtance; the ligamentary ſubſtance was pretty compleat, as was the cord, and the placenta adhered to the ſuperior part of the uterus with conſiderable firmneſs.

The whole courſe of the inteſtinal canal was in a highly diſeaſed ſtate; but our time being ſhort, and the particular object of our ſearch obtained, we did not proceed further in enquiry.

A ſtrict attention has been paid to relate only ſimple facts. A miſtake with reſpect to its being extra-uterine is impoſſible, from the care taken in examination; it is therefore brought forward as a proof of the reality of ſuperfœtation. The time of her being taken with pains in the firſt inſtance, exactly correſponds with the ſize of the fœtus retained after the expulſion of the ſubſequent conception.

The pain ſhe felt in a day or two after delivery, was exactly in every reſpect like that complained of before, ſo that whatever produced diſeaſe before the ſecond impregnation, was, to her own ſenſations, that which cauſed thoſe under which ſhe now labored, and under which ſhe died; which was evidently a fœtus, enveloped in its own membranes within the cavity of the uterus.

The child is, I believe, now living.

